# Dual-Modality Bioactive Repair of Facial Nerve Injury Using PEG-Mediated Axonal Fusion and Extracellular Vesicles-Enriched Human GMSC Secretome

**DOI:** 10.21203/rs.3.rs-8642995/v1

**Published:** 2026-02-11

**Authors:** Justin C. Burrell, Qunzhou Zhang, Shi Shihong, Mykhailo M. Tatarchuk, David R. Clizbe, D. Kacy Cullen, Anh D. Le

**Affiliations:** University of Pennsylvania; University of Pennsylvania; University of Pennsylvania; University of Pennsylvania; University of Pennsylvania; University of Pennsylvania; University of Pennsylvania

**Keywords:** Polyethylene glycol fusion, Gingiva mesenchymal stem cells, Secretome therapy, Axonal regeneration, Facial nerve repair, Peripheral nerve regeneration

## Abstract

Facial nerve injuries may cause severe functional deficits with limited recovery after standard neurorrhaphy. Polyethylene glycol (PEG)–mediated axonal fusion enables immediate structural reconnection and prevents Wallerian degeneration, while gingiva-derived mesenchymal stem cell (GMSC) secretome provides a potent, acellular trophic stimulus that supports neuronal survival and remyelination. We report a dual-modality bioactive repair interface integrating PEG fusion with human GMSC secretome to accelerate neural repair. Fluorescently labeled secretome components were rapidly internalized by neurons *in vitro* within 24 hours. Also, GMSC secretome preserved axonal structure in a dose-depedent manner in a rat sciatic nerves *ex vivo*. In a rat facial nerve transection model, PEG fusion restored immediate compound muscle action potentials (CMAPs) and, at 7 days post-repair, PEG fusion and GMSC secretome preserved large-caliber myelinated axons. In particular, secretome treatment increased axon density and myelination, while the combined PEG + secretome approach produced the highest axon density and axonal area. By 42 days, combined treatment yielded the greatest total axon and myelinated axon densities, organized myelin architecture, and superior functional recovery, reflected by increased CMAP amplitudes. These findings define a clinically translatable, cell-free repair platform that couples immediate axonal fusion with trophic signaling to promote rapid and durable recovery after cranial and peripheral nerve injury.

## Introduction

Peripheral nerve injuries (PNIs) involving the facial nerve often result in devastating functional and psychosocial deficits, with current microsurgical repair approaches offering limited recovery. Standard neurorrhaphy depends on slow axonal regeneration—typically 1–3 mm per day—leading to prolonged denervation, target atrophy, and incomplete reinnervation. Thus, strategies capable of both preserving existing axons and accelerating regeneration are urgently needed to improve outcomes following facial nerve transection.

Among emerging repair modalities, polyethylene glycol (PEG)–mediated axonal fusion represents a fundamentally different approach to nerve preservation. Rather than relying solely on regrowth, PEG fusion physically reconnects severed axonal membranes, immediately restoring electrophysiological continuity and preventing the initiation of Wallerian degeneration. This intervention stabilizes axonal architecture, maintains distal target viability, and enables rapid functional recovery. Recent preclinical and clinical studies have demonstrated robust axon plasmalemmal and electrophysiological restoration within minutes after fusion and meaningful behavioral improvements across multiple peripheral nerve models.^1–9^ PEG-fusion technology has now advanced into several ongoing clinical trials, including an upcoming phase 3 evaluation, underscoring the potential clinical impact of this strategy for acute nerve repair.

Gingiva-derived mesenchymal stem cell (GMSC) secretome provides a compelling biological adjunct to this structural repair platform. Derived from neural crest–origin MSCs, the GMSC secretome is rich in neurotrophic, angiogenic, and immunomodulatory factors, including BDNF, GDNF, VEGF, and IL-10. Prior work has demonstrated that GMSC secretome and its exosomal fractions promote Schwann-cell migration, axonal outgrowth, and remyelination in both peripheral and cranial nerve models.^10–15^ Importantly, the cell-free nature of secretome facilitates a standardized, off-the-shelf deployment alongside surgical repair.

Here, we hypothesize that combining PEG-mediated axonal fusion with GMSC-derived secretome will synergistically enhance both early axonal preservation and long-term regeneration following facial nerve transection. Using a combination of *in vitro*, *ex vivo*, and *in vivo* rat models, we evaluated this combinatorial strategy across acute (7-day) and chronic (42-day) time points, assessing electrophysiological recovery, axonal regeneration, remyelination, and fiber-size distributions. This work defines a dual-modality framework that integrates structural reconnection with paracrine bioactivity to achieve rapid and sustained neural repair.

## Materials and Methods

### Animals

All animal procedures were reviewed and approved by the Institutional Animal Care and Use Committee (IACUC) of the University of Pennsylvania and were conducted in accordance with the National Institutes of Health Guide for the Care and Use of Laboratory Animals and applicable institutional guidelines. Female Sprague–Dawley rats aged 6 to 8 weeks were housed in polycarbonate cages with two animals per cage in a temperature-controlled animal facility maintained at 23°C ± 2°C, 40–65% relative humidity, and a 12-hour light/dark cycle. Animals were provided a standard laboratory diet and allowed ad libitum access to drinking water. All efforts were made to minimize animal suffering and to reduce the number of animals used.

### Culture of human gingiva-derived mesenchymal stem cells (GMSCs)

Primary GMSCs were routinely isolated and cultured in our lab.^14, 16^ Gingival tissues were obtained as remnants of discarded tissues from healthy donors who received a dental procedure following informed consents approved by the Institutional Review Board (IRB) at University of Pennsylvania. GMSCs were cultured at 37°C in a humidified tissue-culture incubator with 5% CO_2_ in the complete growth medium: α-minimum essential medium (α-MEM) supplemented with 10% FBS (fetal bovine serum) (Zen-Bio, Inc., Durham, NC), 100U/ml penicillin and 100μg/ml streptomycin, 2 mM L-glutamine, 1× non-essential amino acid (NEAA), and 55μM β-mercaptoethanol (β-ME) (ThermoFisher Scientific).

### Generation of extracellular vesicle (EV)-enriched secretome from GMSCs cultured under defined xeno-free culture conditions

We recently established an optimized xeno-free induction condition under which GMSCs produce secretome significantly enriched with extracellular vesicles (EVs) and soluble factors.^17^ In brief, GMSCs were seeded into tissue-treated culture (TC) dishes (100-mm) precoated with 20μg/mL of poly-L-ornithine (PLO) at a cell density of 2 · 10^6^ cells/dish and cultured with 10mL of regular complete α-MEM growth medium. 24h later, cells were washed with PBS for three times and continuously cultured with 10mL of xeno-free induction medium composed of DMEM-low glucose (LG)/Ham F12 (2:1) supplemented with 1% antibiotics, transferrin-depleted N2 (1), NEAA (1), 55μM β-ME (all from ThermoFisher Scientific), 10 ng/mL of epidermal growth factor (EGF), 10 ng/mL of basic fibroblast growth factor (bFGF) (Peprotech), and 5μM SB431542 (Cayman Chemicals).^17^ After cultured for 3 days, cells were harvested by trypsinization (0.05% Trypsin/EDTA), seeded into new PLO-precoated dishes (100-mm), and continuously cultured for another 3 days with the xeno-free induction medium.

Subsequently, the conditioned medium was harvested and centrifuged at 1000×g for 20 mina at 4°C to remove cells and large debris. The supernatant was collected and filtered through a syringe filter with a 0.22μm hydrophilic PVDF membrane (Thermos Fisher Scientific). Then, the Ultracel^®^−10 KDa Amicon^®^ Ultra-15 Centrifugal Filters (Merk Millipore Inc.) were utilized to further concentrate the pre-processed conditioned medium by about 100-folds (e.g. from 10mL to 0.1mL) through ultrafiltration followed by washing twice with 10mL of PBS to obtain the final concentrated secretome product.^17, 18^ The protein concentration of secretomes was determined using the Pierce^™^ BCA Protein Assay kit (Thermo Scientific). The secretome product was aliquoted and stored at − 80°C for further use.

### Nanoparticle tracking analysis (NTA) of EVs in GMSC-derived secretome

The concentration and size (diameter) of extracellular vesicles in GMSC-derived secretome were analyzed using the Spectradyne’s nCS1 Nanoparticle Analyzer at the Extracellular Vesicle (EV) Core Facility of University of Pennsylvania School of Veterinary Medicine (UPenn Vet EVC).

### Uptake of EVs in GMSC-derived secretome by cultured neurons

Primary cortical neurons were isolated from embryonic day 18 (E18) rat fetuses. Following maternal euthanasia, fetuses were transferred to cold Hanks’ balanced salt solution (HBSS), and cerebral cortices were microdissected under a stereomicroscope. Tissue was enzymatically dissociated in 0.25% trypsin + 1 mM EDTA at 37°C for 10–12 min, followed by digestion with 0.15 mg/mL DNase I. Dissociated cells were centrifuged at 3000 rpm for 3 min, resuspended in Neurobasal medium + B27 + GlutaMAX + 1% penicillin-streptomycin, and plated on poly-D-lysine-coated 24-well plates. For EV uptake assay, GMSC-derived secretome samples were labeled with the PKH26 Red Fluorescent Cell Linker Kit (Sigma-Aldrich, St. Louis, MO) according to the manufacturer’s protocol. The prelabeled samples were applied to Amicon→Ultra-centrifugal Filters (Ultracel-10K) and centrifuged to remove the unbound dye, followed by washing twice with 0.5 ml PBS and then reconstituted in 100μl PBS. Rat cortical neurons were seeded in 8-well chamber slide (2·10^4^/well) and cultured for 72. Then PKH26-labeled GMSC-derived EVs were added into cells (10μg/ml) and incubated at 37°C for 24h. Cultures were fixed with 4% paraformaldehyde and immunostained for βIII-tubulin (TUJ1) and images were captured with confocal microscopy.

### Western blotting analysis

To assess the enrichment of EV protein markers in concentrated secretomes, equal amount of each concentrated secretome sample (20 μg of total protein) was mixed with Laemmli loading buffer and boiled at 95°C for 5 min. The denatured samples were separated on 12% sodium dodecyl sulfate (SDS)-polyacrylamide gel and electroblotted onto Amersham^™^ Protran^™^ Nitrocellulose Blotting Membrane (Cat#10600002; Cytiva). After blocking with 5% nonfat dry milk in 1×Tris-buffered saline containing 0.1% Tween-20 (TBST), the membrane was incubated at 4°C overnight with primary antibodies, including CD9 (20597–1-AP, 1:1000, Proteintech), CD63 (25682–1-AP, 1:1000, Proteintech), CD81 (66866–1-Ig, 1:1000; ProteinTech), and α-syntenin 1 (ab133267; 1:1000; Abcam). Following incubation with a corresponding horseradish peroxidase (HRP)-conjugated secondary antibodies at room temperature for 1h, blot signals were developed with ECL^™^ Western Blotting Detect Reagents (GE Health Care) and images were captured using Amersham Imager 680 (GE Health Care Life Sciences).^17^

#### Ex Vivo Sciatic Nerve Explant Culture.

Sciatic nerves were harvested from adult Sprague–Dawley rats under sterile conditions immediately following euthanasia. Nerve segments were trimmed to uniform lengths and placed in 24-well plates with cell-culture inserts containing Neurobasal^™^ medium supplemented with B27, GlutaMAX^™^, and penicillin–streptomycin. Cultures were maintained at 37°C in a 5% CO_2_ incubator. Explants were randomly assigned to three groups: (1) control (medium only), (2) GMSC-derived secretome at 25 μg/mL, and (3) GMSC-derived secretome at 50 μg/mL. The secretome was lyophilized, stored at − 80°C, and reconstituted in sterile water immediately before use. Media were replenished every 48 hours.

#### Nerve Explant Morphometric Analysis.

Explant tissues were fixed in 10% neutral-buffered formalin, cryoprotected overnight in 30% sucrose, embedded in OCT compound (Tissue-Tek), and snap-frozen in a dry-ice/isopentane slurry. Serial 20 μm axial cryosections were collected on a cryostat and mounted on Superfrost Plus^™^ slides. Sections were immunolabeled with anti-mouse SMI31/32 (BioLegend) for neurofilaments followed by AlexaFluor 594-conjugated secondary antibody. Confocal images were acquired at consistent magnification and laser power using a Nikon A1R microscope.

#### Facial Nerve Fusion and Electrophysiological Assessment.

Facial nerve injury was induced in adult rats by microsurgical transection of the buccal branch followed by neurorrhaphy with or without PEG-mediated axonal fusion. The fusion protocol was performed as previously described: the transected nerve ends were bathed in calcium-free Plasmalyte, treated with methylene blue, exposed to PEG 3350 (50% diluted in sterile water) for 2 minutes, rinsed with calcium-containing lactated Ringer’s solution, and sealed with fibrin glue—with or without GMSC secretome. Immediate compound muscle action potentials (CMAPs) were recorded from the vibrissae pad using subdermal electrodes (1 Hz; 0–2 mA, 0.1 ms biphasic pulse; 20–3000 Hz band-pass filter) on a Natus electrodiagnostic system. Animals exhibiting positive CMAPs were enrolled in one of four post-fusion treatment groups: (1) Neurorrhaphy + Fusion + Secretome, (2) Neurorrhaphy + Fusion, (3) Neurorrhaphy + Secretome, or (4) Neurorrhaphy only. Secretome was applied topically in fibrin at 50 μg/mL.

#### Long-Term Electrophysiological Recording.

At 42 days post-injury, CMAPs were recorded both proximally and distally to the repair site. Stimulation was applied proximal to the lesion (proximal CMAP) or at the main trunk (distal CMAP), and responses were recorded from the vibrissae pad. CMAP amplitude was calculated as the peak-to-baseline voltage averaged across ≥ 5 waveforms.

#### Histological Analysis.

At 7 and 42 days post-repair, nerves were harvested, embedded in OCT, and cryosectioned (20 μm) for immunohistochemistry with SMI31/32 (neurofilament) and MBP (myelin basic protein). Confocal z-stacks were acquired using a 20× air or 60× oil objective. Fiji software was used for image processing: channels were separated, axons thresholded, and segmented using size (0.2–70 μm^2^) and circularity (0.15–1.00) parameters. Morphometric parameters included axon area (%), axon count, and axon diameter (converted from measured area). Four non-overlapping 175 × 175 μm ROIs were analyzed per section. At least 100 fibers per animal were quantified for axon-diameter distribution.

### Statistical Analysis.

All analyses were performed by a blinded investigator using coded datasets. Values are reported as mean ± SEM. Immediate CMAP data were analyzed with a two-tailed Mann–Whitney U test. Two-way ANOVA with Tukey’s post hoc test was used for multi-group comparisons. Significance was set at *p* < 0.05.

## Results

### GMSCs produce EV-enriched secretome under the defined xeno-free induction condition

**Figure 1:**
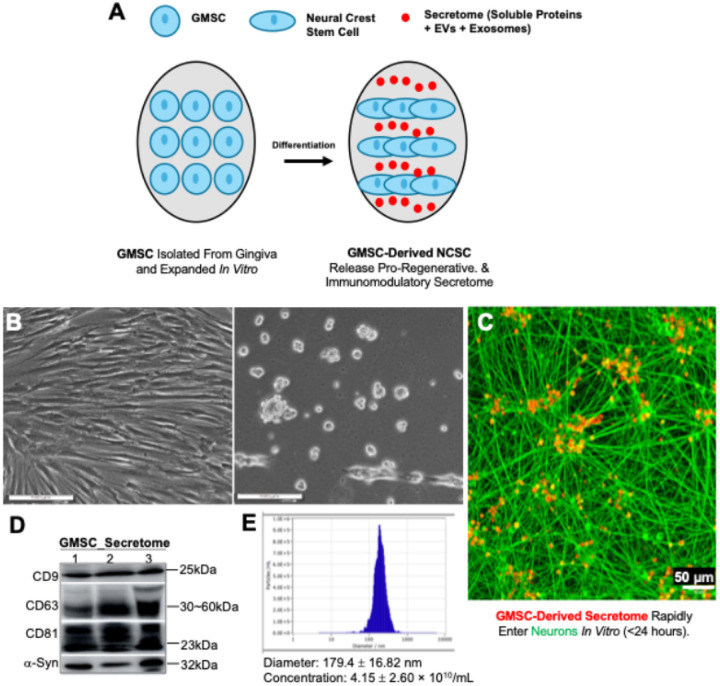
Defined Xeno-Free GMSC Induction Produces an EV-Enriched Neurotrophic Secretome. **(A)** Schematic illustrating the differentiation of gingiva-derived mesenchymal stem cells (GMSCs) into neural crest stem-cell–like (NCSC) cells that secrete pro-regenerative and immunomodulatory factors. **(B)** Phase-contrast micrographs showing morphological transition of GMSCs from fibroblast-like to NCSC phenotype following 6 days of induction under xeno-free conditions. Scale bar = 100 μm. **(C)** Confocal image demonstrating rapid uptake of PKH26-labeled GMSC secretome (red) by cultured rat cortical neurons immunolabeled for βIII-tubulin (green). Scale bar = 50 μm **(D)** Western blot showing enriched expression of canonical extracellular-vesicle markers (CD9, CD63, CD81, a-Syn) in the GMSC secretome. **(E)** Nanoparticle characterization of the secretome (Spectradyne nCS1), showing a monodisperse population of extracellular vesicles with a mean diameter of 179.4 ± 16.82 nm and peak concentration of 4.15 ± 2.60 × 10^10^ particles/mL, consistent with an exosome-dominated profile.

We recently reported that GMSCs cultured under an optimized xeno-free induction culture condition were converted into neural crest stem-like cells with enhanced pro-nerve regeneration potentials (Fig. 1A).^14^ Most recently, we showed that GMSCs under the similar induction condition produced secretome significantly enriched with extracellular vesicles (EVs) and soluble factors and were conferred with enhanced regenerative effects on rat tongue defect model.^17^ Herein, we also showed that GMSCs under the defined xeno-free culture condition for 6 days underwent remarkable morphological changes (Fig. 1B). To confirm that the gingiva mesenchymal stem cell (GMSC)–derived secretome is directly internalized by neurons, fluorescently labeled secretome was applied to primary rat cortical neurons in culture (Fig. 1C). Within 24 hours of exposure, robust intracellular fluorescence was observed throughout the neuronal soma and extending along βIII-tubulin–positive neurites, indicating rapid uptake and intracellular transport. Confocal microscopy revealed punctate and vesicular accumulation within both axonal shafts and growth cones, suggesting endocytic or receptor-mediated internalization rather than nonspecific adsorption to the plasma membrane. Co-localization analysis with TUJ1 (βIII-tubulin) confirmed the cytosolic and neuritic distribution of internalized material. Quantitatively, more than 85% of neurons displayed detectable fluorescent signal following 24 h incubation, with intensity plateauing thereafter, consistent with a saturable uptake mechanism. These data confirm that the human GMSC secretome is efficiently internalized by neurons within 24 hours, providing mechanistic support for its rapid neuroprotective and regenerative effects observed *in vivo*. Consistent with our recent study^17^, a remarkable enrichment in the expression of a panel of EV protein markers, including CD9, CD63, CD81, and α-syntenin 1, was observed in GMSC-derived secretome under this induction condition as determined by Western blot (Fig. 1D). Nanoparticle tracking analysis (NTA) showed that enriched EV particles in GMSC-derived secretome have an average diameter of 179.4 ± 16.82 nm and a concentration of 4.15 ± 2.60 × 10^10^ particles/mL, consistent with an exosome-dominated profile. (Fig. 1E).

#### Secretome Reduces Explant Counts and Delays Axon Fragmentation.

**Figure 2:**
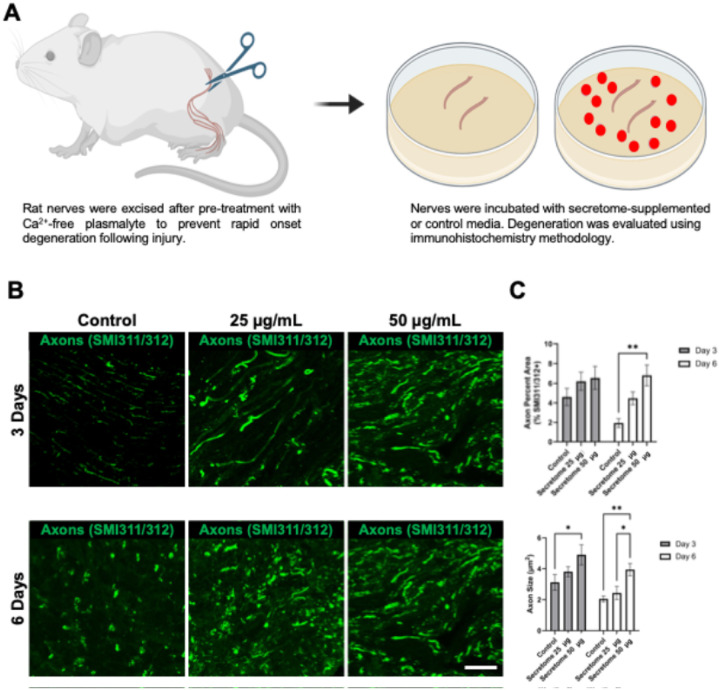
GMSC Secretome Preserves Axonal Integrity in an Ex Vivo Nerve Degeneration Model. **(A)** Sciatic nerves are harvested from adult rats and cultured ex vivo with or without GMSC-derived secretome to assess its effect on axonal preservation and degeneration over time. **(B)** Representative confocal micrographs of rat sciatic nerve explants cultured for 6 days, sectioned longitudinally, and immunostained for neurofilaments (SMI31/32; green). Treatment groups include: control (media only), 25 μg/mL GMSC secretome, and 50 μg/mL GMSC secretome. Scale bar: 50 μm. **(C)**Quantification of axonal area (%Area) and axon size at days 3 and 6 shows that secretome-treated groups—particularly the 50 μg/mL dose—significantly preserved axonal structure in a dose-dependent manner compared to control. Bars represent mean ± SEM (*p < 0.05, **p < 0.01).

Axon fragmentation was evaluated in a sciatic nerve ex vivo model (Fig. 2). At day 3, control explants showed the highest counts (671.6 ± 78.2), significantly greater than 25 mg (444.2 ± 61.9) and 50 mg (412.5 ± 61.7) secretome groups (p < 0.05). By day 6, only the 50 mg group remained significantly lower than control (236.0 ± 12.3 vs. 449.3 ± 65.2; p < 0.05). Secretome 50 mg significantly increased axon size compared to control at both time points (p < 0.01), and %Area was significantly higher at day 6 (6.81 ± 1.05% vs. 1.94 ± 0.45%; p < 0.01). Axon size was also significantly greater in the 50 mg group at both day 3 (4.90 ± 0.65 mm^2^ vs. 3.12 ± 0.51 mm^2^; p < 0.05) and day 6 (3.94 ± 0.40 mm^2^ vs. 2.05 ± 0.19 mm^2^; p < 0.01).

#### PEG Fusion Enables Immediate Functional Reconnection Following Facial Nerve Neurorrhaphy.

**Figure 3:**
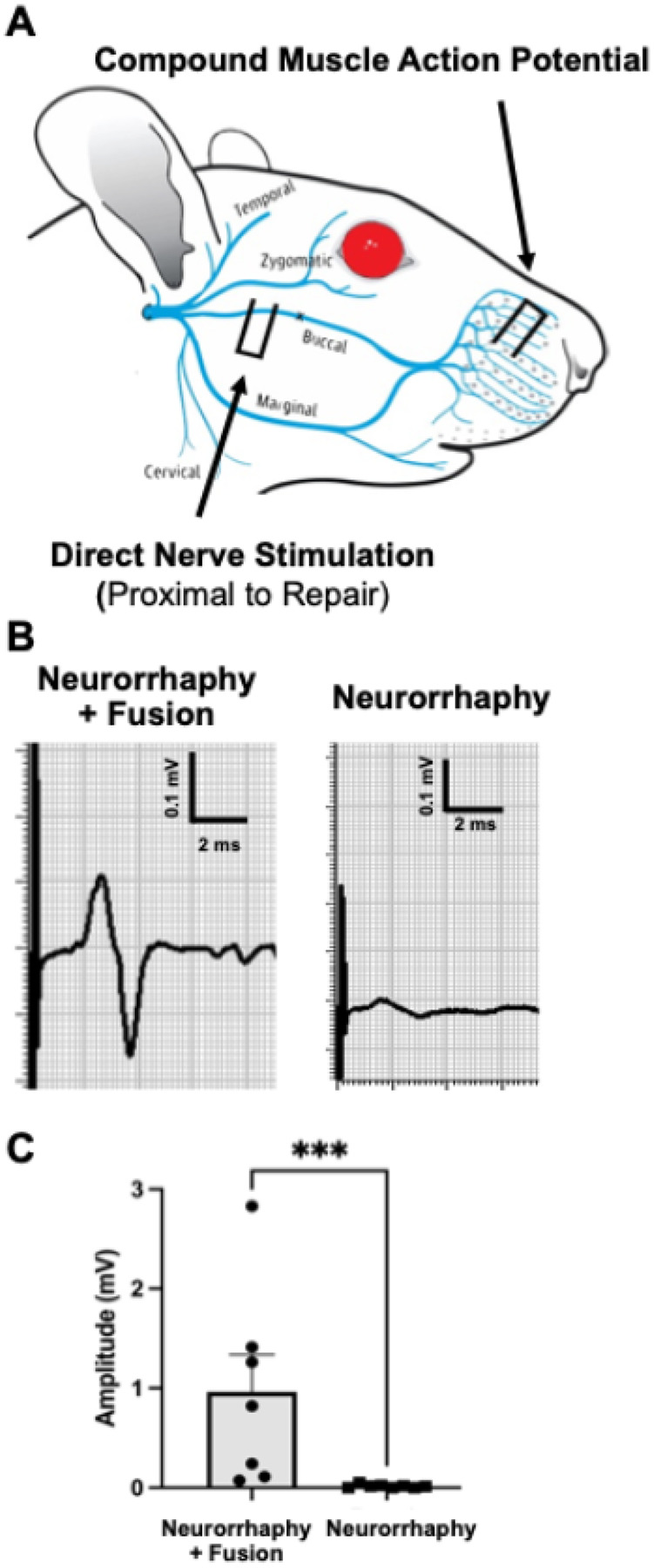
PEG Fusion Enables Rapid Restoration of Evoked Muscle Activity Following Facial Nerve Injury. **(A)** Schematic of rat facial nerve anatomy, illustrating buccal branch transection at the site marked by an ‘X’. Stimulating electrodes are placed proximal to the injury, and compound muscle action potentials (CMAPs) are recorded from the vibrissae pad using subdermal electrodes. **(B)** Representative CMAP traces recorded immediately post-repair. PEG fusion (left) elicits a robust muscle response, while no-fusion controls (right) show minimal activity. **(C)** Quantification of immediate CMAP amplitude demonstrates significantly greater evoked response in PEG-fused animals compared to non-fused controls (p < 0.001, Mann–Whitney U test). Each dot represents an individual animal; bars show mean ± SEM.

PEG fusion produced immediate CMAP recovery (0.96 ± 0.37 mV), significantly greater than no-fusion controls (0.015 ± 0.006 mV; p = 0.0003), confirming effective reconnection (Fig. 3). Secretome was applied only after successful fusion confirmation.

#### Acute (7 Days) Response: PEG Fusion Preserves Large Axons While Secretome Accelerates Regeneration Following Facial Nerve Neurorrhaphy.

**Figure 4:**
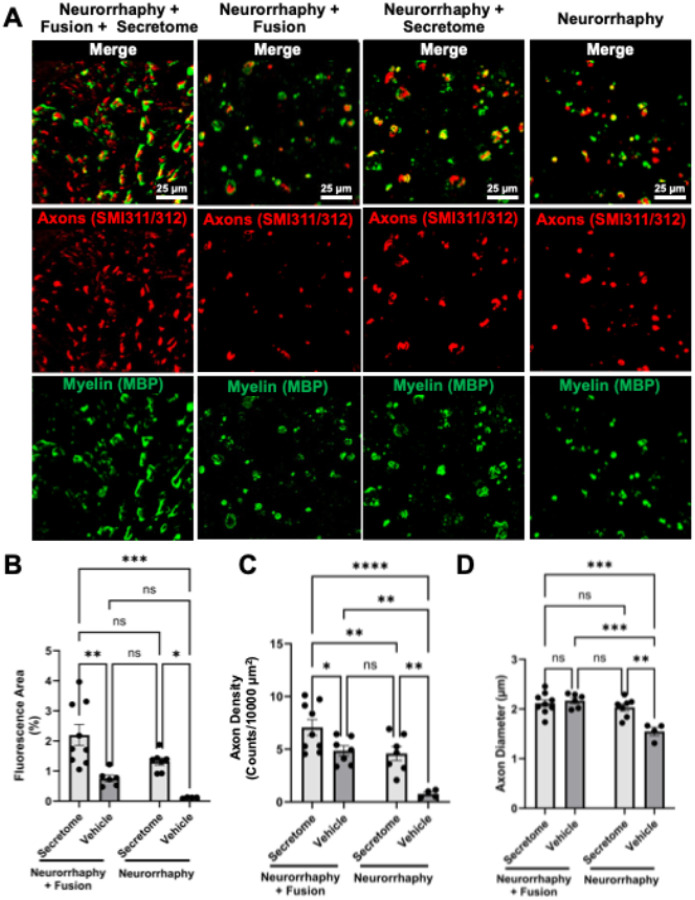
PEG Fusion and GMSC Secretome Promote Axonal Regeneration and Preserve Large-Caliber Fibers. **(A)** Representative confocal micrographs of distal facial nerve cross-sections immunostained for axons (SMI31/32; red) and myelin (MBP; green) at 7 days post-repair. Treatment groups include Neurorrhaphy + Vehicle, Neurorrhaphy + Fusion + Vehicle, Neurorrhaphy + Secretome, and Neurorrhaphy + Fusion + Secretome. Scale bar = 25 μm. Large, intact myelinated axons were clearly visible following Neurorrhaphy + Fusion, whereas Neurorrhaphy alone contained few or degenerating myelinated profiles indicative of early Wallerian degeneration. In Neurorrhaphy + Secretome, smaller myelinated fibers were observed, though it remains unclear whether these represent preserved axons or accelerated remyelination of regenerating fibers. **(B–D)** Quantification of axonal fluorescence area (%), axon density (axons/1000 μm^2^), and mean axon diameter (μm). Both Fusion and Secretome independently increased axonal regeneration relative to Vehicle (p < 0.05–0.0001), producing an additive enhancement when combined. All parameters were analyzed using two-way ANOVA with Tukey’s post-hoc test (*p < 0.05, **p < 0.01, ***p < 0.001, ****p < 0.0001). Data represent mean ± SEM; n = 4–9 nerves per group.

Large, intact myelinated axons were clearly visible following Neurorrhaphy + Fusion, whereas Neurorrhaphy alone contained few or degenerating myelinated profiles, indicative of early Wallerian degeneration (Fig. 4). In Neurorrhaphy + Secretome, smaller myelinated fibers were observed, though it remains unclear whether these represent preserved axons or accelerated remyelination of regenerating fibers.

Quantitatively, axon fluorescence area (%) showed significant main effects of Fusion (*F*(1, 22) = 7.85, *p* = 0.0104) and Secretome (*F*(1, 22) = 23.63, *p* < 0.0001). Mean values were 2.20 ± 0.35 (Neurorrhaphy + Fusion + Secretome; n = 9), 0.76 ± 0.11 (Neurorrhaphy + Fusion + Vehicle; n = 6), 1.32 ± 0.12 (Neurorrhaphy + Secretome; n = 7), and 0.12 ± 0.01 (Neurorrhaphy + Vehicle; n = 4), indicating additive enhancement of early axonal regeneration.

Axon density (axons / 1000 μm^2^) likewise revealed strong effects of Fusion (*F*(1, 23) = 21.58, *p* = 0.0001) and Secretome (*F*(1, 23) = 21.98, *p* = 0.0001) with no interaction (*F*(1, 23) = 0.98, *p* = 0.33). Densities were 7.07 ± 0.76 (Neurorrhaphy + Fusion + Secretome; n = 9), 4.85 ± 0.48 (Neurorrhaphy + Fusion + Vehicle; n = 7), 4.60 ± 0.66 (Neurorrhaphy + Secretome; n = 7), and 0.76 ± 0.18 (Neurorrhaphy + Vehicle; n = 4).

Mean axon diameter (μm) demonstrated significant interaction (*F*(1, 22) = 12.41, *p* = 0.0019) and main effects of Fusion (*F*(1, 22) = 21.26, *p* = 0.0001) and Secretome (*F*(1, 22) = 7.98, *p* = 0.0099). Diameters were 2.12 ± 0.07 (Neurorrhaphy + Fusion + Secretome; n = 9), 2.17 ± 0.06 (Neurorrhaphy + Fusion + Vehicle; n = 6), 2.03 ± 0.07 (Neurorrhaphy + Secretome; n = 7), and 1.55 ± 0.08 (Neurorrhaphy + Vehicle; n = 4).

Together, these findings show that PEG fusion rapidly preserves large-caliber fibers, while the GMSC secretome promotes early axonal outgrowth and remyelination, yielding the greatest regenerative area and axon density within one week.

#### Chronic (42-Day) Outcome: Combined PEG Fusion and Secretome Treatment Promotes Sustained Axonal Regeneration, Remyelination, and Functional Recovery Following Facial Nerve Neurorrhaphy.

**Figure 5:**
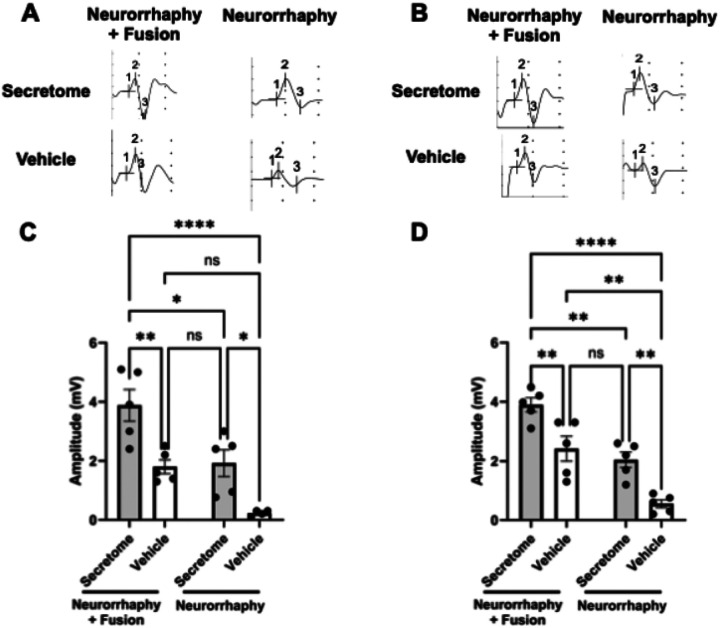
Additive Enhancement of Functional Recovery by PEG Fusion and GMSC Secretome at 42 Days Post-Repair **(A–B)** Representative compound muscle action potential (CMAP) traces recorded from the proximal and distal facial nerve at 42 days post-repair. Treatment groups include Neurorrhaphy + Vehicle, Neurorrhaphy + Fusion + Vehicle, Neurorrhaphy + Secretome, and Neurorrhaphy + Fusion + Secretome. **(C–D)** Quantification of proximal and distal CMAP amplitudes (mV) demonstrates significant main effects of both Fusion and Secretome (p = 0.0004–<0.0001) without interaction, indicating additive improvement in evoked muscle responses. Mean amplitudes were highest in the Neurorrhaphy + Fusion + Secretome group, with each treatment alone providing partial recovery relative to Vehicle. All data represent mean ± SEM; n = 4–5 nerves per group; two-way ANOVA with Tukey’s post-hoc test (*p < 0.05, **p < 0.01, ***p < 0.001, ****p < 0.0001).

The early regenerative effects translated into substantial long-term improvements in axonal integrity and function. At 42 days, both fusion groups and the non-fused Secretome group exhibited marked enhancement in electrophysiological muscle activity (Fig. 5). Proximal CMAP amplitudes demonstrated significant main effects of Fusion (F(1, 15) = 20.29, p = 0.0004) and Secretome (F(1, 15) = 23.19, p = 0.0002), with no interaction (F(1, 15) = 0.24, p = 0.63). Mean amplitudes were highest in the Neurorrhaphy + Fusion + Secretome group (3.88 ± 0.53 mV; n = 5) compared with Fusion + Vehicle (1.80 ± 0.23 mV), Secretome alone (1.92 ± 0.45 mV), and Vehicle controls (0.23 ± 0.05 mV). Distal CMAP amplitudes followed a similar pattern with strong main effects of Fusion (F(1, 16) = 43.61, p < 0.0001) and Secretome (F(1, 16) = 27.64, p < 0.0001), yielding 3.90 ± 0.24, 2.42 ± 0.42, 2.04 ± 0.26, and 0.55 ± 0.13 mV, respectively. These findings confirm additive improvements in neuromuscular conduction across both fusion and secretome treatments.

**Figure 6:**
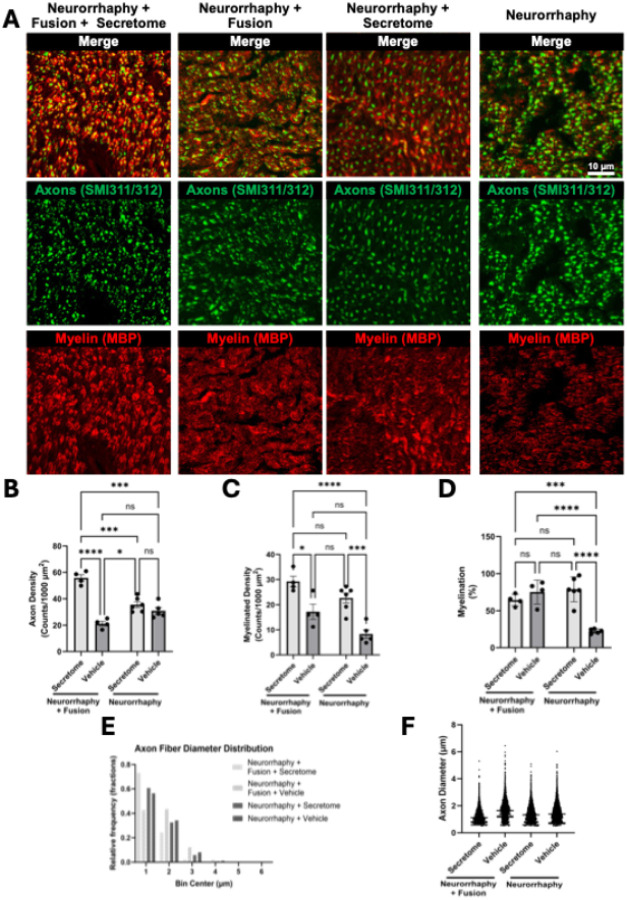
GMSC Secretome Augments Axonal Density and Myelination 42 Days After PEG Fusion. **(A)** Representative confocal micrographs of repaired nerve cross-sections immunolabeled for axons (SMI311/312; green) and myelin (MBP; red) in Neurorrhaphy + Fusion + Secretome, Neurorrhaphy + Fusion, Neurorrhaphy + Secretome, and Neurorrhaphy groups at 42 days post-repair. Merged images **\**reveal dense, axons with myelin organization in both fusion groups and in the non-fused Secretome group compared to standard repair. Scale bar = 10 μm. **(B)** Axon density, **(C)** myelinated axon density, and **(D)** percent myelination were all significantly greater in Neurorrhaphy + Fusion + Secretome, Neurorrhaphy + Fusion, and Neurorrhaphy + Secretome groups compared to Neurorrhaphy alone, indicating additive effects of fusion and secretome treatment. **(E)** Histogram of axon diameter distribution and **(F)** violin plot of axon diameters revealed a leftward shift in the Fusion + Secretome group, suggesting an increased population of small-caliber regenerating axons alongside preservation of overall axonal content. Data are presented as mean ± SEM; Axon density and myelinated axon density calculated from 175×175 μm ROIs. *p < 0.05, **p < 0.01, ***p < 0.001, ****p < 0.0001 by two-way ANOVA with Tukey’s post-hoc test.

Corresponding histological analyses revealed dense axonal regeneration and organized myelin in all treated groups, contrasting with faint, fragmented MBP labeling and scattered degenerating fibers in standard neurorrhaphy (Fig. 6). The combined treatment produced the highest axon density (55.75 ± 2.48 axons/1000 μm^2^; n = 4), significantly exceeding Fusion + Vehicle (23.66 ± 2.97), Secretome alone (35.55 ± 2.18), and Vehicle controls (34.98 ± 3.44) (Secretome main effect: F(1, 15) = 33.02, p < 0.0001; interaction: F(1, 15) = 30.76, p < 0.0001).

Myelinated-fiber density displayed additive effects of Fusion (F(1, 15) = 12.06, p = 0.0034) and Secretome (F(1, 15) = 35.25, p < 0.0001), reaching 29.2 ± 2.10 (Neurorrhaphy + Fusion + Secretome), 17.2 ± 3.02, 22.67 ± 2.00, and 8.32 ± 1.71 axons/1000 μm^2^ across the respective groups. Percent myelination similarly showed significant main effects (Fusion: F(1, 15) = 10.47, p = 0.0055; Secretome: F(1, 15) = 14.85, p = 0.0016; interaction: F(1, 15) = 32.47, p < 0.0001), reaching 64.25 ± 3.93, 75.15 ± 8.21, 78.80 ± 6.93, and 22.36 ± 2.64% of total axons, respectively—confirming robust remyelination in treated nerves.

Axon-diameter histograms (Fig. 6; Table 1) revealed right-skewed profiles dominated by small-caliber regenerating fibers (1–2 μm), particularly in the Neurorrhaphy + Fusion + Secretome group (73.2% of fibers ≈ 1 μm). Broader distributions in the Fusion + Vehicle group suggested partial preservation of large fibers. Mean diameters were 1.23 ± 0.01, 1.42 ± 0.01, 1.71 ± 0.01, and 1.52 ± 0.01 μm across groups, with significant differences among all distributions (D = 0.13–0.31, p < 0.0001). Skewness (0.94–1.17) confirmed small-fiber dominance, while kurtosis (1.18–2.17) reflected active remodeling rather than static preservation.

Collectively, these data indicate that PEG fusion re-establishes axonal continuity and preserves large-caliber fibers, while the GMSC secretome supports trophic, regenerative, and remyelinating processes. Their combination yielded the greatest axon density, balanced small- and large-fiber populations, and near-complete restoration of motor conduction by 42 days post-repair.

## Discussion

**Figure 7:**
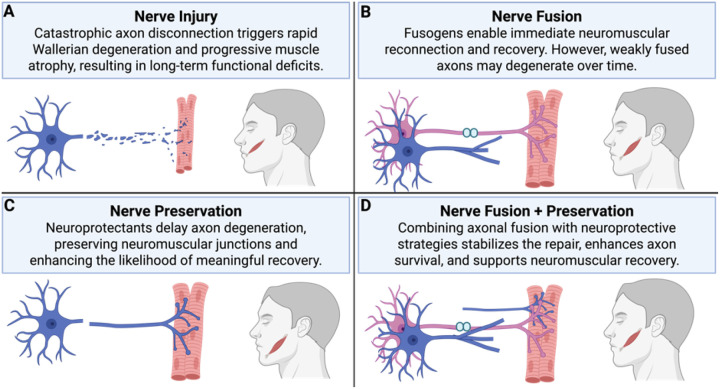
Conceptual Overview of Therapeutic Strategies for Enhancing Nerve Repair. **A. Nerve Injury:** Axonal transection leads to catastrophic distal axon degeneration and rapid muscle atrophy, compromising functional recovery. **B. Nerve Fusion:** PEG-mediated axonal fusion rapidly reconnects severed axonal membranes, restoring immediate conduction and initiating regenerative plasticity. However, weakly fused axons may still degenerate without additional support. **C. Nerve Preservation:** Neuroprotective interventions prevent distal axon degeneration, preserving neuromuscular junctions and increasing the likelihood of meaningful regeneration. **D. Nerve Fusion + Preservation**: Combining fusion with neuroprotective strategies stabilizes reconnection, enhances axon survival, and promotes long-term functional restoration. Together, these mechanisms support our novel combined strategy: **Secretome-Augmented Nerve Fusion**, a dual-modality approach that merges structural repair with molecular support to maximize early and sustained neuromuscular recovery.

This study demonstrates that combining PEG-mediated axonal fusion with a human gingiva mesenchymal stem cell (GMSC) secretome provides complementary and synergistic mechanisms for enhancing facial nerve repair. Using a rat facial-nerve model, we show that PEG fusion acutely preserves large-caliber axons and delays Wallerian degeneration, while the GMSC secretome promotes sustained axonal regeneration, remyelination, and functional recovery. Together, this dual-modality approach addresses both the immediate structural discontinuity caused by transection and the longer-term need for a trophic and redox-stabilizing microenvironment that sustains repair (Fig. 7).

PEG-mediated fusion rapidly restored axonal continuity, as confirmed by immediate compound muscle action potential (CMAP) recovery. This acute reconnection likely reflects resealing of disrupted axolemma and restoration of ionic conductance across fused axons, consistent with prior PEG-fusion studies in peripheral and central systems.^1–9^ Recent mechanistic work has linked this membrane resealing to ferroptosis-associated lipid remodeling, wherein redox-regulated stabilization through GPX4 and FSP1 activity is essential for successful fusion.^19^ The present data—showing preserved large myelinated axons—are consistent with suppression of lipid peroxidation and ferroptotic degeneration.

The addition of GMSC secretome amplified regenerative outcomes across both time points. Fluorescent labeling confirmed that secretome components were rapidly internalized by cortical neurons within 24 hours, localizing to soma and neurites. This finding suggests an active uptake process that could directly influence neuronal metabolism, cytoskeletal organization, and redox status. Functionally, secretome-treated nerves exhibited higher axon densities and myelinated-fiber counts, consistent with enhanced axonal extension and remyelination, while coupling biochemical rescue to structural repair.

By 7 days post-repair, the combination of PEG fusion and GMSC secretome produced the greatest axon density and fluorescence area, indicating both preserved and newly sprouting fibers. Although smaller-caliber myelinated axons predominated in secretome-treated nerves, this pattern likely represents regenerating rather than degenerating fibers—a finding supported by prior work showing that GMSC secretome and exosomes induce Schwann-cell repair phenotypes, upregulate neurofilament and MBP expression, and accelerate myelin regeneration.^10, 12–15^ Consistent with these findings, our results suggest that GMSC-derived trophic and exosomal factors enhance Schwann-cell–neuron crosstalk, promoting synchronized axonal extension and remyelination while amplifying the neuroprotective benefits initiated by PEG-mediated fusion. Notably, a related study demonstrated that intramuscular transplantation of tonsil-derived MSC–Schwann-like cells delayed axonal degeneration, preserved neuromuscular junctions, and improved reinnervation after peripheral nerve repairm^20^, reinforcing the concept that MSC-based biologics can delay Wallerian degeneration and maintain distal target viability—effects that parallel PEG fusion’s early preservation phase.

The neuroprotective and pro-regenerative effects observed here are also consistent with evidence that transplanted human GMSCs promote dopaminergic neuron survival and motor recovery in Parkinson’s disease models by suppressing oxidative stress, stabilizing mitochondrial membrane potential, and activating the STAT3–BCL-2/BAX axis.^21^ Transcriptomic and proteomic analyses from that study revealed that GMSCs upregulate neuronal differentiation and axonogenesis genes while suppressing metabolic and inflammatory pathways—mechanistically validating our interpretation that secretome delivery recapitulates these cytoprotective pathways as a cell-free treatment.

By 42 days, the pro-regenerative and maturation effects were both sustained and amplified. Nerves treated with PEG + secretome exhibited the highest overall axon and myelinated-fiber densities, accompanied by superior myelin organization and balanced axon-caliber distributions. Axon-diameter histograms revealed right-skewed profiles dominated by small regenerating fibers, particularly in the combination group, consistent with ongoing regeneration. Importantly, the coexistence of small regenerating and mature large-caliber axons indicates that fusion-mediated preservation and secretome-mediated regeneration act in parallel rather than sequentially, creating a continuum from immediate reconnection to sustained regrowth. These morphologic outcomes translated into the highest proximal and distal CMAP amplitudes, demonstrating that restored conduction reflects genuine reinnervation rather than residual signaling through spared fibers.

Mechanistically, this synergy likely reflects the convergence of two complementary repair modalities: PEG fusion re-establishes axonal continuity, stabilizes membranes, and preserves cytoskeletal integrity, while the GMSC secretome creates a pro-regenerative, anti-inflammatory microenvironment that supports Schwann-cell migration, angiogenesis, and axon elongation. The additive—and in several parameters, synergistic—interactions between these mechanisms produced the greatest axon density, myelination, and functional recovery across all time points.

From a translational perspective, both components of this strategy are advancing toward clinical readiness. PEG fusion has entered active human trials, including multiple phase 2 studies and a phase 3 program in preparation, while secretome-based biologics are showing promising safety and efficacy in early clinical use. A recent systematic review of patient data concluded that stem-cell–derived secretomes enhance axonal continuity and functional recovery after nerve injury with excellent safety profiles.^22^ These findings underscore the feasibility of combining acellular trophic biologics with intraoperative fusion techniques to create a scalable, off-the-shelf repair paradigm.

Limitations of this study include the 42-day observation period, which does not capture late-stage myelination or behavioral recovery, and the use of a single transection model. Ongoing work will evaluate delayed-repair scenarios, large-animal translation, and proteomic dissection of the GMSC secretome to identify dominant trophic mediators driving regeneration and remyelination.

## Conclusion

PEG-mediated axon fusion combined with GMSC-derived secretome produced complementary and synergistic effects on facial nerve repair. PEG fusion preserved large-caliber axons and enabled immediate electrophysiological reconnection, while the secretome enhanced axonal regeneration, myelination, and functional recovery. At 7 days, the combination achieved the highest axon density and area coverage, reflecting early neuroprotection and regeneration; by 42 days, it produced the greatest restoration of muscle activity and organized myelinated fiber architecture.

Together, these findings support a clinically translatable paradigm that unites structural reconnection with trophic bioactivity to accelerate and sustain recovery after nerve injury. This dual-modality approach has broad potential for improving outcomes across cranial and peripheral nerve repair contexts.

## Supplementary Material

Supplementary Files

This is a list of supplementary files associated with this preprint. Click to download.

- SupplementalFigureWBGMSCSecretomeuncropped.pdf

## Figures and Tables

**Table 1:** 42 Day Axon Fiber Diameters (Cumulative Percentage)

Axon Diameter Bin Center	Fusion + Secretome	Fusion + Vehicle	Secretome	Vehicle
1 μm	73.20	42.59	60.77	56.40
2 μm	24.37	43.27	32.54	34.22
3 μm	2.24	12.05	5.92	8.12
4 μm	0.14	1.77	0.71	1.09
5 μm	0.04	0.22	0.06	0.16
6 μm	0.00	0.10	0.00	0.02

## Data Availability

All data supporting the findings of this study are available from the corresponding author upon reasonable request. Data are stored in institutional repositories and will be shared in accordance with applicable ethical approvals, material transfer agreements, and institutional policies.

## Highlights

- PEG-mediated axonal fusion immediately restores continuity and prevents Wallerian degeneration.
- Human GMSC-derived secretome rapidly enters neurons and enhances axonal regeneration and remyelination.
- Combined PEG fusion and secretome treatment yields highest axon density and functional recovery in vivo.
- Establishes a clinically translatable, cell-free bioactive interface for cranial and peripheral nerve repair.
